# Mal de Debarquement Syndrome: A Matter of Loops?

**DOI:** 10.3389/fneur.2020.576860

**Published:** 2020-11-10

**Authors:** Viviana Mucci, Iole Indovina, Cherylea J. Browne, Franco Blanchini, Giulia Giordano, Lucio Marinelli, Bruno Burlando

**Affiliations:** ^1^School of Science, Western Sydney University, Penrith, NSW, Australia; ^2^Laboratory of Neuromotor Physiology, Istituto di Ricovero e Cura a Carattere Scientifico Fondazione Santa Lucia, Rome, Italy; ^3^Department of Biomedical and Dental Sciences and Morphofunctional Imaging, University of Messina, Messina, Italy; ^4^Translational Neuroscience Facility, School of Medical Sciences, UNSW Sydney, Sydney, NSW, Australia; ^5^Department of Mathematics, Computer Science and Physics, University of Udine, Udine, Italy; ^6^Department of Industrial Engineering, University of Trento, Trento, Italy; ^7^Department of Neuroscience, Rehabilitation, Ophthalmology, Genetics, Maternal and Child Health (DINOGMI), University of Genova, Genova, Italy; ^8^Division of Clinical Neurophysiology, Department of Neurosciences, Istituto di Ricovero e Cura a Carattere Scientifico (IRCCS) Ospedale Policlinico San Martino, Genova, Italy; ^9^Department of Pharmacy, University of Genova, Genova, Italy

**Keywords:** mal de debarquement syndrome, brain derived neurotrophic factor, calcitonin gene related peptide, functional loops, synaptic plasticity, systems and control theory

## Abstract

**Introduction:** Mal de Debarquement Syndrome (MdDS) is a poorly understood neurological disorder affecting mostly perimenopausal women. MdDS has been hypothesized to be a maladaptation of the vestibulo-ocular reflex, a neuroplasticity disorder, and a consequence of neurochemical imbalances and hormonal changes. Our hypothesis considers elements from these theories, but presents a novel approach based on the analysis of functional loops, according to Systems and Control Theory.

**Hypothesis:** MdDS is characterized by a persistent sensation of self-motion, usually occurring after sea travels. We assume the existence of a neuronal mechanism acting as an oscillator, i.e., an adaptive internal model, that may be able to cancel a sinusoidal disturbance of posture experienced aboard, due to wave motion. Thereafter, we identify this mechanism as a multi-loop neural network that spans between vestibular nuclei and the flocculonodular lobe of the cerebellum. We demonstrate that this loop system has a tendency to oscillate, which increases with increasing strength of neuronal connections. Therefore, we hypothesize that synaptic plasticity, specifically long-term potentiation, may play a role in making these oscillations poorly damped. Finally, we assume that the neuromodulator Calcitonin Gene-Related Peptide, which is modulated in perimenopausal women, exacerbates this process thus rendering the transition irreversible and consequently leading to MdDS.

**Conclusion and Validation:** The concept of an oscillator that becomes noxiously permanent can be used as a model for MdDS, given a high correlation between patients with MdDS and sea travels involving undulating passive motion, and an alleviation of symptoms when patients are re-exposed to similar passive motion. The mechanism could be further investigated utilizing posturography tests to evaluate if subjective perception of motion matches with objective postural instability. Neurochemical imbalances that would render individuals more susceptible to developing MdDS could be investigated through hormonal profile screening. Alterations in the connections between vestibular nuclei and cerebellum, notably GABAergic fibers, could be explored by neuroimaging techniques as well as transcranial magnetic stimulation. If our hypothesis were tested and verified, optimal targets for MdDS treatment could be found within both the neural networks and biochemical factors that are deemed to play a fundamental role in loop functioning and synaptic plasticity.

## Introduction

Mal de Debarquement Syndrome is a condition characterized by a subjective sensation of self-motion (i.e., rocking, swaying, bobbing), which persists after an initial exposure to passive motion, usually after sea travel but occasionally after air or overland trips. Commonly, many people report this condition in its temporary form, “*Mal de Debarquement”* (MdD), with symptoms usually subsiding within 48 h, or in the worse cases a few weeks (1). However, a small subset of these individuals do not recover, and experience chronic symptoms for months, up to years, after the initial onset due to passive motion exposure, thus developing “*Mal de Debarquement Syndrome”* (MdDS) (2). The prevalence of the syndrome in the population is currently unknown, while a neurotology clinic survey reported that 1.3% of patients were diagnosed annually (3). Despite the lack of precise epidemiological data, MdDS is considered a rare pathological condition with associated psychosocial and economic impacts (4). In addition to its most recognized primary symptoms (i.e., chronic self-motion perception and postural instability), there are a series of secondary symptoms such as brain fog, migraine, visual sensitivity, and associated mood disorders (4). The underlying pathophysiology is still not clear; consequently, there are limited therapeutic and experimental options. In addition to this, low awareness regarding MdDS in the medical community has contributed to high misdiagnosis rates (4, 5), which potentially increases the perceived rarity of the condition.

Emerging research has concluded that the typical contemporaneous MdDS patient is female (though MdDS has been reported in males, the current female to male ratio is 8:2) (6), in the 5–6th decade of life, having been exposed to passive motion, usually cruise ship travels (7). As mentioned, the onset cause of the condition, in a typical MdDS patient is related to the exposure to passive motion and symptoms began after disembarking; those triggered in this way are termed Motion-Triggered (MT) MdDS patients. Interestingly, a similar symptom profile can also be present in individuals that cannot attribute their symptom onset to a passive motion exposure, but rather to a non-motion trigger (non-motion triggered onset MdDS). These cases without any clear trigger are also referred to as Spontaneous Onset (SO) MdDS (4). The classification of SO MdDS remains under review. SO MdDS may be re-classified to come under another central vestibular disorder called Persistent Postural-Perceptual Dizziness (PPPD) (8), given that these two disorders present overlapping symptoms. The distinguish feature so far identified between SO-MdDS and PPPD is that individuals with PPPD do not report the typical temporal relief of symptoms described by patients with MdDS when re-exposed to passive motion (*e.g., being passenger in a driving vehicle*). Thus, this partial and temporal alleviation from symptoms when re-exposed to passive motion has now been described as a key feature in identifying MdDS patients (4). Thus, more research is needed to assess the possibility that SO MdDS and MT MdDS include similar symptom manifestation of differing underlying pathophysiological mechanisms. The theory presented in this manuscript will solely focus on MT MdDS, indicated hereafter as “MdDS” in this manuscript.

A series of hypotheses have been formulated to explain the pathogenesis of this condition. It has been proposed that MdDS is the result of Vestibular Ocular Reflex (VOR) maladaptation (9), involving velocity storage (VS), a central vestibular mechanism that increases the time constant of the VOR with respect to that of semicircular canal (SCC) afferents. In this hypothesis, the authors propose that a cross-axis-coupled stimulus (e.g., roll while pitching, a type of stimulation that can be experienced by passengers on a boat) may alter the VS of the VOR (9). The VS circuit is thought to be located in vestibular-only (VO) neurons, which are found in the medial and superior vestibular nuclei (VN) of the brainstem (10), and has been investigated in non-human primates (11). This study demonstrated that monkeys without VS, and thus having a very short VOR time constant, did not develop abnormal responses to roll while rotating. A similar mechanism was hypothesized to be present in humans (9). VO neurons are γ-aminobutyric acid (GABA) neurons and their axons decussate in the brainstem, where information is then projected to the reticulospinal and vestibulospinal pathways (12).

Cohen and colleagues proposed that the cause for the appearance of MdDS symptoms could potentially be a maladaptive response to the typical oscillatory frequencies experienced during air or sea navigation, which ranges between 0.2 and 0.3 Hz, and activates the lower limbs into compensatory rocking and swaying movements for balance maintenance (13). In MdDS, VO neurons, on both sides of the brainstem, are theorized to persistently oscillate at these frequencies after disembarking (9), and these oscillations may have originated in the nodulus of the vestibulocerebellum, which has control over the VS (9, 13, 14). Indeed, such activity was observed in the nodulus of the rabbit (15), suggesting that a similar mechanism may be possible and present in humans. Moreover, additional symptoms which are a part of MdDS manifestations, such as brain fog, anxiety, depression, and sensitivity to sound and light, may be the result of the inability to “turn off” these incessant oscillations (9). Cortical changes also seem to contribute to MdDS (16). In particular it has been theorized that MdDS is a disorder of the central mechanism that generates a memory for an internal representation of passive movement (17). Accordingly, in MdDS patients a decrease in functional connectivity has been reported in different brain regions, including visual-vestibular processing areas (e.g., middle temporal visual area [V5]), the brain's default mode network (that includes the cingulate cortex), somatosensory network (including the postcentral gyrus), and central executive network including the dorsolateral prefrontal cortex (2, 17, 18). Resting-state functional Magnetic Resonance Imaging (fMRI) studies have also shown variations in functional connectivity involving the left entorhinal cortex (EC)/amygdala, with increased connectivity to posterior visual and vestibular processing areas, and decreased connectivity to multiple prefrontal areas (17). Also, high-density Electroencephalogram (EEG) studies have shown that MdDS patients have a higher synchronicity during periods of higher symptom severity, specifically in vestibular projections to the limbic system (1, 19). While abnormalities in the limbic system are correlated to abnormal motion perception (17, 18), the EC is known to also play a role in keeping the hippocampus active during sleep for memory consolidation (20). This has been hypothesized as why in some MdDS patients symptoms present after a night's sleep and not immediately after landing/disembarking (2, 16). Following this hypothesis, a series of experimental treatment protocols have been developed to treat MdDS sufferers with the use of neuromodulation techniques targeting these regions (21, 22).

In addition to neutrally-centered hypotheses, which may not be mutually exclusive, a new hypothesis was formulated which proposed that gonadal hormones may influence MdDS pathophysiology (7). Correlations between MdDS and hormonal factors have been reported, driven by the fact that MdDS patients are mostly females and that the average onset age matches with the perimenopausal phase (23). It is known that hormones play an important role in various vestibular pathologies such as vestibular migraine, and Meniere's disease (24), and that there are correlations between hormonal fluctuations and various inner ear symptoms such as vertigo, instability, tinnitus, hearing loss and intra-aural pressure (25). Additionally, it is well-known from animal studies and human clinical data that hormonal changes also influence neurochemical pathways that are linked to depression (26). As for migrainous patients, a recent pilot study showed that pregnant MdDS patients reported an alleviation of symptoms during the first two trimesters (27). Following these preliminary observations, hormones were theorized to play a role in aggravating patient symptoms as well as in rendering an individual more susceptible to developing the disorder *per se* (7, 23). Specifically, it was theorized that patients who developed MdDS may have had, at the time when onset occurred, significant decreases in estrogen levels which altered their GABAergic system, as well as Calcitonin Gene-Related Peptide (CGRP) levels (7). Recently, CGRP has been implicated in the pathophysiology of migraine and depression, which are also comorbidity factors of MdDS (2, 7, 28). It is known to support vestibular function and, more specifically, to strengthen the VOR (29, 30). Accordingly, CGRP positive neurons have been found in VN and the vestibulocerebellum (30). In addition, CGPR could be overlooked for its role in neuroplasticity, e.g., influencing neurotransmitters such as the brain-derived neurotrophic factor (BDNF) (31, 32). Despite the above hypotheses, knowledge about the comprehensive mechanisms of MdDS is still lacking, thereby hindering the possibility of developing resolutive treatments for the condition. Therefore, this manuscript aims to combine the relevant aspects and ideas from these theories and review them within a theoretical model based on **Systems and Control Theory**. This is an interdisciplinary field combining mathematics and engineering to study the functioning and the emergent behavior of systems arising both in nature and in engineering. Although most of its subsequent developments are aimed at designing and managing human-made systems, such as processes and machines, the discipline was originally inspired by the study of living processes and is particularly well-suited to model and analyse phenomena in physiology and biology (33). One of the main topics of Systems and Control Theory is the study of feedback loops that accomplish a specific function. Accordingly, our hypothesis provides a pathophysiological mechanism of MdDS involving the interaction of functional loops at various levels, including neural networks and intracellular biochemical pathways.

## The Hypothesis

Our model is based on the hypothesis that, to ensure adaptation to an external oscillatory stimulus, an internal oscillatory behaviors must be activated by a neural network (34). The internal generation of oscillatory behaviors most likely relies on a loop-based arrangement, due to the presence of negative feedback loops containing inhibitory interactions (35). Specifically, our hypothesis relies on **Systems and Control Theory**, whose mathematical formalism was mainly developed in engineering but has been widely applied to biological systems since its origin (33, 36). Following this Theory, a perfect adaptation to an external periodic perturbation (*like a wave*), can only occur thanks to the activation of an “internal model” that cancels the perturbation. In our case, the internal model is a neuronal oscillator that generates a signal of the same type but opposing the forcing input (37). According to this fundamental principle, it could be hypothesized that what has been previously described as the presence of a brain oscillator in MdDS may be part of this mechanism (2). Hence, MdDS could be the pathological permanence of such a compensatory mechanism, after the external perturbation has vanished.

However, no clear evidence is available about the neural site of this hypothetical oscillator. In the first theory described previously, MdDS pathophysiology is attributed primarily to the VN in the brainstem, receiving input from SCC and generating vestibulo-ocular, vestibulocollic, vestibulospinal, and vestibulo-thalamo-cortical pathways (13). As already mentioned, it has been proposed that MdDS is driven by an oscillation between VO neurons on each side of the brainstem at frequencies of 0.2–0.3 Hz, controlled by output from cerebellar nodular neurons (13, 38). Interestingly, similar oscillatory behavior has been experimentally induced in the vestibulocerebellum of rabbits through a rolling about the longitudinal axis (15). In these experiments, 5% of climbing fibers in the uvula and nodulus started firing periodically at the same frequency after the sinusoidal vestibular stimulation had stopped, persisting for 200–300 s (15). Although obtained in animal studies, these results were believed to provide a potential neural basis for oscillations at 0.2 and 0.3 Hz manifesting as rocking, swaying, and bobbing in MdDS patients (13, 14, 39).

Besides identifying a brain oscillator, in order to understand the pathogenesis of MdDS a mechanism converting the adaptation to environmental oscillations into a permanent noxious condition must be found. According to Systems and Control Theory, positive loops are a common distinctive feature of multi-stationary systems that can undergo transitions among different equilibrium points (35, 40). These kinds of transitions are thought to operate also in processes of pathogenesis, and therefore, the identification of a positive loop could be a key step in the understanding of MdDS onset. As shown below, we identify this multi-stationary positive loop with an intracellular biochemical pathway involved in synaptic rearrangement.

### Biomechanical Analysis

In order to formulate a hypothesis regarding the mechanisms implicated in MdDS pathophysiology, we first consider a biomechanical analysis of body posture from the standpoint of **Systems and Control Theory**.

**Figure 1:**
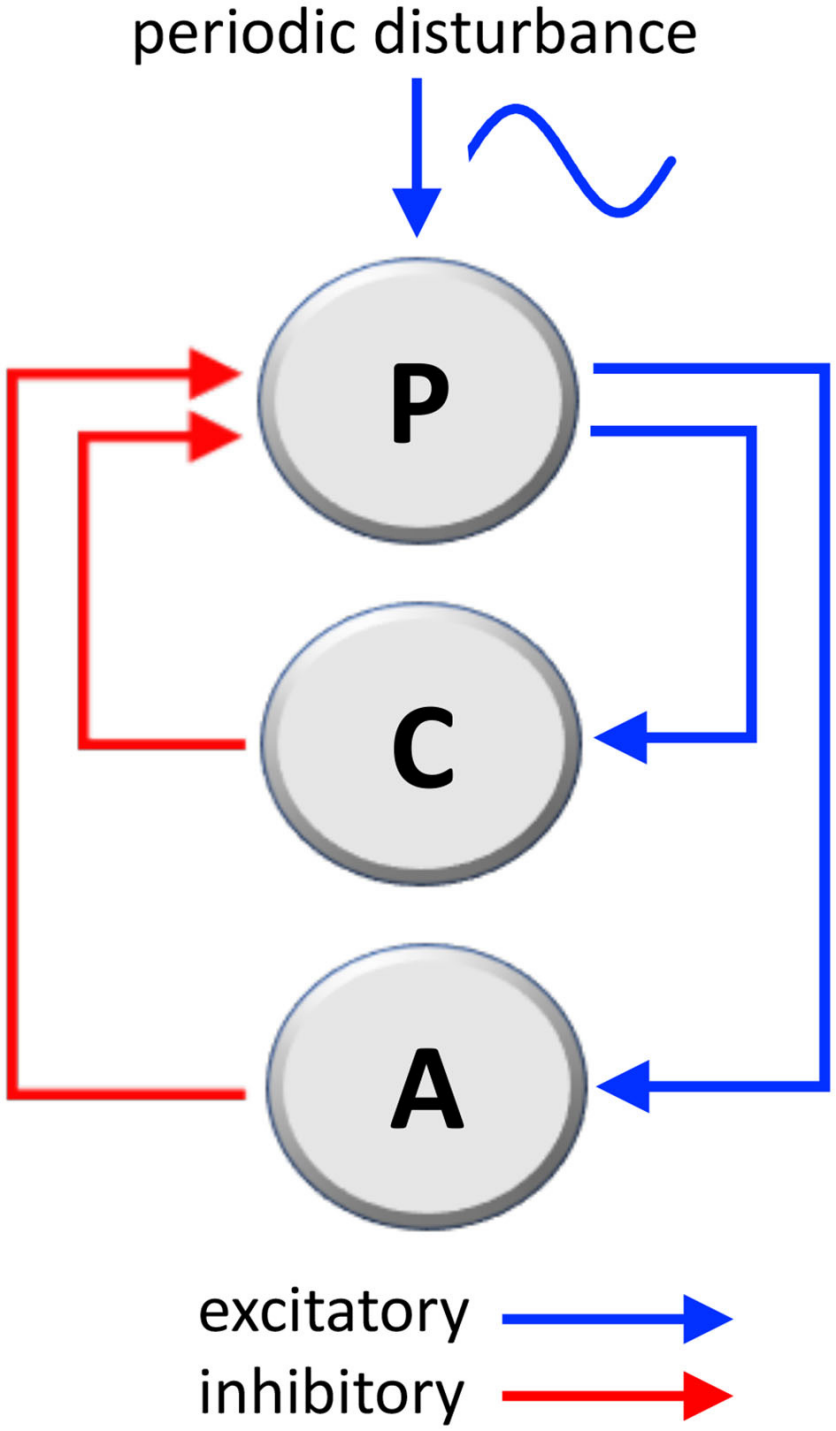
Mechanical loops involved in the stabilization of body's posture and the adaptation to a periodic disturbance, such as passive environmental movements experienced by travelers aboard. P, posture mechanism; C, posture-stabilizing feedback control; A, adaptation mechanism (C and A are negative feedback loops; see Supplementary Materials for a mathematical analysis).

The considered mechanical system that governs body posture, labeled as **P** in Figure 1, is composed of two loops:

- One, labeled as **C** in Figure 1, is the well-studied **stabilizing posture control mechanism** (41, 42), whose function is of ensuring the correct angle (the angle between main body axis and gravity axis).
- The second, labeled as **A** in Figure 1, which we consider as the **adaptation mechanism**, has the function of adapting the posture in the presence of a persistent sinusoidal disturbance (wave motion, craft fluctuations, or similar).

Both the stabilization loop **C** and the adaptation loop **A** are **negative loops** (having one inhibitory step) because their action is in **opposition**, respectively, to destabilizing gravity and to the external disturbance signal. The following considerations are based on well-known physical laws.

In addition to this, in order to fully understand the loop theory, few more points must be considered:

- The **stabilization loop C** must compensate the discrepancies between the position, the posture angle, and the angular speed, namely the derivative of the angle. For doing this, the stabilization loop requires a feedback of the proportional-derivative type, which compensates for both posture and angle errors and their derivatives.
- The **adaptation loop A** is activated to ensure perfect adaptation. For example, it is able to cancel a persistent disturbance whenever it comes into play.
- **Perfect (or semi-perfect) adaptation** requires the generation of a signal **that cancels the** external disturbance, hence it needs to have the same frequency as the disturbance, but needs to be in phase opposition, so that the sum of the two signals is almost zero.
- To be synchronized with the disturbance (in particular, in phase opposition), the compensating signal must be generated by a **feedback loop**, otherwise it is impossible to robustly generate a signal of the same frequency and synchronization.
- To achieve perfect adaptation, the **adaptation loop** must therefore include an oscillator capable of producing a signal having the same frequency as the external disturbance, an assertion supported by the Internal Model Principle (37, 43). Remarkably, the idea of an internal model has been invoked also for MdDS and firstly mentioned by Hain and Helminski (44).

Within our hypothesis, if the external signal is removed (disembarking), then the oscillator in the adaptation loop **A** could remain active for a time under normal conditions, or for longer time under pathological status (when developing MdDS). This oscillator is not capable of destabilizing the posture, since the main stabilizing loop **C** prevails. Yet, the effect of the oscillator persists in generating phantom sensations, possibly at a frequency very close to the initial forcing disturbance. This phenomenon manifests in the patients as a sensation of self-motion (*bobbing, swaying and rocking*). The mathematical description of the above loop system can be found as Supplementary Materials.

### Physiological Analysis

We consider now the possibility of establishing a link between the **mechanical loop system** of Figure 1 and **vestibular neural networks**, by using a neuroanatomical representation.

In the mechanical loop system, the **stabilization loop C** can be easily matched to a postural reflex that corrects postural bias. This element can be identified with vestibulocollic (VCR) and vestibulospinal (VSR) reflexes that are realized by VN and induce compensatory movements maintaining head and postural stability and preventing falls (45). The VCR and VSR involve SCC, otolith, neck proprioception, and visual afferences to the VN, where they become processed through commissural inhibition and ipsilateral integration and filtering (46, 47). One mechanism involved in the processing and fine-tuning of the final reflexes is the VS mechanism, which is particularly relevant when considering head rotation movements (angular acceleration). This mechanism is believed to prolong the SCC afferent signal, by extending the VOR time constant with respect to the SCC signal time constant, and then improving compensatory responses to low-frequency rotations of the head (48, 49). Similarly to this, there is another mechanism (gravity estimator) which processes linear acceleration movements, by integrating SCC and otolith inputs, and estimates head tilt (50). Both mechanisms act as integrators provided with negative loops avoiding error accumulation due to afferent signal noise (50).

A correspondence between these mechanisms and vestibular pathways can be found in the scheme proposed by Galiana and Outerbridge for bilateral VOR pathways in the cat (51). This scheme shows a commissural inhibitory circuit between the two contralateral VN, which is connected on each side to two ipsilateral circuits spanning between VN and the cerebellum. According to the viewpoint of Systems and Control Theory, the central commissural circuit is a double negative loop, i.e., it has an even number of inhibitory steps (two) and therefore it is a candidate multi-stationary system with at least two stable equilibrium points. The two lateral elements are negative loops, i.e., they have an odd number of inhibitory steps (one) and therefore they are candidate oscillators (35, 40). These circuits could be at least partially overlapped with vestibular networks that are regarded to be relevant in MdDS pathophysiology, such as VOR and VS. Specifically, the external negative loops could correspond to the noise correction mechanisms described for the integrators of SCC and otolith afferences, while it is known that cutting the commissural fibers connecting the contralateral VN permanently destroys the VS mechanism (52).

**Figure 2:**
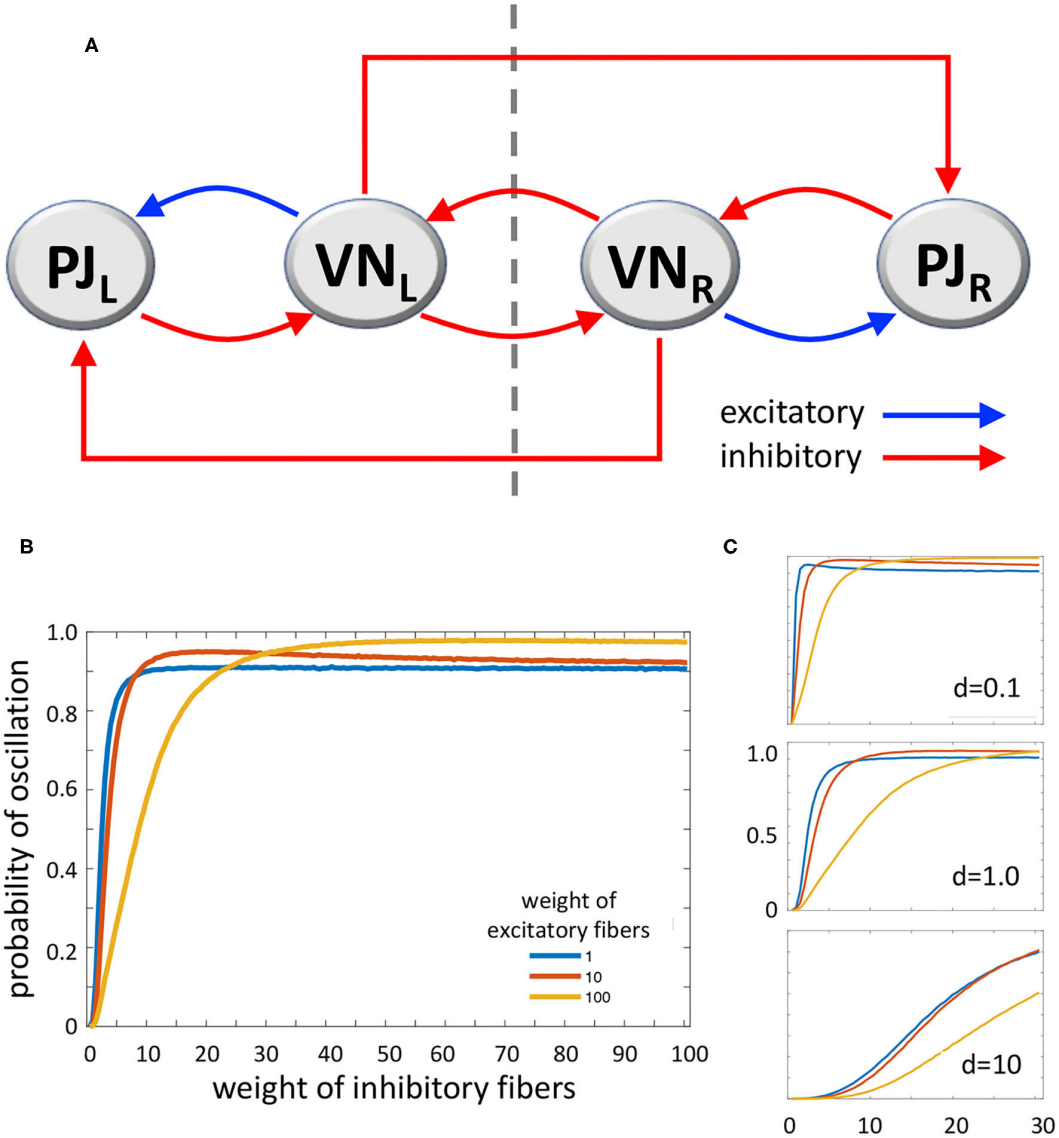
**(A)** Diagram of the loop system envisaged for vestibulocerebellar neural circuits, consisting of two ipsilateral negative loops, a central, contralateral, double-negative loop, and two contralateral vestibulocerebellar connections. PJ, Purkinje cell; VN, vestibular nuclei; R and L, subscripts refer to right and left sides of the brainstem; dashed line, brainstem midline. **(B)** MatLab analysis of the above system yielding the probability of oscillation (i.e., the fraction of Jacobian matrices associated to the loop system with positive-real-part complex eigenvalues, out of 100,000 randomly generated Jacobian matrices). The curves represent the variation of oscillation probability when the weight of inhibitory connections varies in the range 1–100, in arbitrary units, and the weight of excitatory connections is 1 (blue curve), 10 (red curve), and 100 (yellow curve). The weight of self-inhibitory connections (matrix diagonal entries) is always set to 1. **(C)** Probability of oscillation calculated as above, when the weight of inhibitory connections varies in the range 1–30, the weight of excitatory connections is 1 (blue), 10 (red), and 100 (yellow), and the weight of self-inhibitory connections (d) is 0.1 (top), 1.0 (middle), and 10 (bottom). Plot axes as in B.

According to Systems and Control Theory, a model which includes a **triple loop chain** with a core positive loop flanked by two negative loops could generate oscillations upon alternate stimulation of the two sides. The two lateral negative loops, behaving as oscillators, would induce the central loop to toggle between its two stable states (53) (Figure 2A). However, VN also project as GABAergic fibers to the inferior olive that in turn projects as glutamatergic climbing fibers to the contralateral cerebellar flocculonodular lobe, altogether realizing contralateral inhibitory pathways (54). Mossy fibers are also derived from VN to cerebellum, which could participate to the network; however, experimental recording of periodical firing at the same frequency of sinusoidal vestibular stimulation was recorded in climbing fibers (see above) (15). The complete system (Figure 2A), consisting of the triple loop discussed above with the further addition of two contralateral inhibitory connections, can still produce oscillations, depending on the values of the parameters that regulate the interactions among the elements of the system. Therefore, a main role in the tendency to oscillate could be played by the relative strength of excitatory and inhibitory pathways.

In order to evaluate the capability of this system to yield sustained oscillations, we studied it by using MatLab (*The MathWorks, Inc., Natick, MA, USA*). We computed the oscillation probability as a function of the strength of excitatory and inhibitory pathways. In particular, given the graph in Figure 2A, with the addition of stabilizing self-inhibitions for each functional agent, we randomly generated instances of the associated Jacobian matrix (see Supplementary Materials):

(1) $$\begin{array}{l} J=\left[\begin{array}{cccc} -\alpha & \mu & -\nu & 0\\ -\kappa_{1} & -\beta & -\sigma & 0\\ 0 & -\varphi & -\gamma & {-\kappa }_{2}\\ 0 & -\tau & \xi & -\delta \end{array}\right] \end{array}$$

where each non-zero entry, denoted by a Greek letter, is generated as a random number with absolute value between 0 and 1, multiplied by a scaling coefficient that can be chosen differently for: (i) diagonal entries associated with self-inhibitory connections (α, β, γ, δ), (ii) entries associated with excitatory connections (μ, ξ), and (iii) entries associated with inhibitory connections (ν, κ_1_, σ, φ, κ_2_, τ), so as to modulate their relative strength. Then, we computed the eigenvalues of each randomly generated Jacobian matrix *J*, to evaluate the fraction of Jacobian matrices having strictly complex eigenvalues with positive real part; the presence of this type of eigenvalues is associated with persistent oscillations. Figure 2B shows the probability of oscillation (*precisely, the fraction of matrices with positive-real-part complex eigenvalues out of 100,000 randomly generated matrices*) when (i) the weight of self-inhibitory connections is set to 1 in arbitrary units, (ii) the weight of inhibitory connections grows from 1 to 100, and (iii) the weight of excitatory connections is chosen as 1 (*blue curve*), 10 (*red curve*) and 100 (*yellow curve*). In Figure 2C the same curves are shown, with the weight of inhibitory connections growing from 1 to 30, and the weight of self-inhibitory connections (*i.e., the matrix diagonal entries*) set to 0.1, 1.0, and 10.

The results of our numerical study show that, if the **strength of inhibitory connections** is low (*i.e. their weight equals about 0–20 in arbitrary units*), the tendency to produce contralateral oscillations is inversely correlated with the strength of excitatory connections. Conversely, if the strength of inhibitory connections is intermediate (*about 20–40*), then the tendency to produce oscillations is high and almost independent of the strength of excitatory connections. Finally, if the **strength of inhibitory connections is high** (*about 40–100*) **the effect of excitatory connections is reversed, since higher values of the latter induce a higher tendency to produce oscillations** (Figure 2B). As for the role of self-inhibitory connections *(i.e., the matrix diagonal entries, associated with weight d*), Figure 2C shows that oscillations are more likely when the strength of these connections is lower (*as expected, given their stabilizing effect*); however, irrespective of their strength, the trend of the curves is always qualitatively similar (*in* *Figure 2C* *plots, the axes and the curve colors have the same meaning as in* *Figure 2B*). Taken together, these data show that our loop system can generate oscillations, and moreover, the tendency to oscillate increases together with the increasing strength of inhibitory connections, reaching its highest when both inhibitory and excitatory connections are strong.

Given its ability to realize oscillations, the loop system depicted in Figure 2A could be hypothesized to be the **adaptation loop A**, i.e., the internal model, of the mechanical loop system (Figure 1). Specifically, during adaptation to external stimulation, the circuit alternatively activates and inhibits VN neurons located on the right and left side of the brainstem. This model of course is a simplified schematic representation of the actual neural networks, while other components could contribute to the adaptive mechanism. For instance, in agreement with studies on the role of efference copy and feed-forward loop in postural adaptation to environmental disturbance (55), a feed-forward loop could help make the system faster and more accurate. However, the feedback loop system is to be considered the key component of the oscillator.

### Active Scenario

Having this basis aforementioned, we are now able to consider the passive motion and environmental stimuli (active scenario). In the presence of an oscillatory movement, where the individual is exposed to passive motion, such as being passenger in a boat or plane, the VN are stimulated from side to side by their various afferents, i.e., vestibular sensors, proprioceptors, and visual inputs. This stimulation corresponds to the sinusoidal external disturbance applied to the mechanical loop system (*Figure 1*). If the **adaptation A (internal model)** circuit were absent, the **stabilization C (postural reflex)** circuit would continuously stimulate the proprioceptors and the muscle tone to correct the posture. However, we assume that the **adaptation loop** becomes entrained by this kind of external stimulation, thereby undergoing an oscillatory behavior that cancels the environmental stimulation and abolishes the need of postural correction. This scenario fits the previously commented assumption that in ship travelers VO neurons are entrained to oscillate at frequencies of about 0.2–0.3 Hz, (under control of cerebellar nodulus neurons) (38). Hence, in this scenario the individual exposed to passive motion is going through natural adaptation and compensatory mechanisms of passive motion.

Thereafter, when the individual is disembarking (returning to a stable environment), the oscillatory external stimulus ceases, and the **adaptation loop** system is expected to stop its compensatory work. However, it can be shown through mathematical analysis (see Supplementary Materials) that the adaptation mechanism has poorly damped oscillations for a reasonably wide range of its parameters, possibly explaining the above-mentioned, temporary MdD condition that can be experienced after a ship travel. Conversely, in MdDS the adaptation loop seems to persist beyond any possible delayed damping, thus becoming a permanent oscillator that provides an undesired input to the **stabilization mechanism**. Consequently, this creates a sensation of postural unbalance (phantom sensation of bobbing, rocking, swaying). This interpretation of MdDS seems confirmed by the notion that patients report a feeling of **phantom motion** when in a stable environment (*e.g., on land*), but they find a temporal relief while re-exposed to passive motion. This would happen by being re-exposed to the dominant frequencies of their postural swaying that match fairly with passive motion (*around 0.2–0.3 Hz*) (4).

Hence, a remaining open question concerns the causes of the over-synchronization affecting the neural network that generates MdDS. The mathematical analysis of our neural loop system shows that the tendency to oscillate increases for increasing strength of inhibitory pathways, while it reaches its maximum when the strength of both inhibitory and excitatory pathways is high. Such a result strongly suggests that the **over-synchronization of the internal oscillator may be found in synaptic plasticity** (56), which can induce variations of connection strength in the neural loop system. Most notably, a typical example of this kind of neuronal rearrangement is long-term potentiation (**LTP)** (57). Synaptic plasticity has been shown to occur in Purkinje cells of the flocculonodular cerebellum, as well as in their interconnected vestibular circuit (58, 59). Hence, it can be hypothesized that LTP may occur in these neurons during passive motion (*e.g., when in/on a vehicle*), due to continuous, alternate stimulations from sensory inputs. The distinction between MdD (*rapid healing)* and MdDS (*recalcitrant healing*), could be due to differences in the strength of synaptic plasticity. Therefore, excess synaptic plasticity could be the essential element that switches a physiological mechanism (*internal model*) into a pathological condition.

The involvement of excessive synaptic plasticity has been reported for various disorders, e.g., diminished LTP and long term depression (LTD) in schizophrenia (60), dopamine-driven synaptic facilitation in drug addiction (61), or unbalanced excitatory and inhibitory stimuli on fusiform cells of the dorsal cochlear nuclei in tinnitus (62). Even closer to MdDS, maladaptive cortical plasticity is involved in the pathophysiology of focal dystonia, such as writer's cramp and spasmodic torticollis, where repetitive sensory input reaches cortical sensory areas that show excessive plastic adaptation characterized by increased motor output, excessive muscle contractions, abnormal postures, and involuntary movements (63–66). Similarly, we suggest that in MdDS abnormal plasticity occurs in vestibular and Purkinje neurons. This is strongly supported by the above results from MatLab computational analysis of the loop system modeling the vestibulocerebellar connections in the brainstem. These findings show that an increase in the strength of inhibitory connections renders the loop more incline to oscillate, suggesting that excess LTP in GABAergic fibers could strengthen the vestibulocerebellar oscillator, thus rendering it recalcitrant to vanish when the environmental stimulus ceases (*stable environment*).

If excess synaptic plasticity is responsible for the insurgence of MdDS, we have now to explain why it occurs in some individuals at a certain time. Taking this into account, we aim at a unifying paradigm between vestibular sensory processing and Systems and Control Theory, according to the approach advocated by Burlando (67). Based on this theoretical view, a transition from health to disorder can be always reconducted to a positive loop showing a bifurcation that creates the possibility of shifting from one steady state equilibrium point to another one. Interestingly, LTP has been described as a shift in gene expression due to the activity of a biomolecular positive loop undergoing bifurcation (68). Moreover, a relevant property of dynamic systems showing bifurcation and bistability is hysteresis, meaning that it is easier to maintain the system in one stable state, or equilibrium point, than to make it jump to another stable state by applying or removing a stimulus. In addition, if the strength of the positive loop increases, the system develops irreversibility, i.e., the impossibility of returning to a former equilibrium point by complete removal of the stimulus (69). It has been shown that different cellular and biomolecular processes can be modeled through bistability and hysteresis (70), while positive feedback loops are starting to be taken into consideration also for mechanisms of pathogenesis (e.g., Alzheimer's' Disease) (71). In our model of adaptation to an oscillating environment, the occurrence of synaptic plasticity in the vestibulocerebellar circuit (*Figures 1*, *2*) could be modulated from normal to excessive by the strength of a biomolecular positive loop, thus determining a variable degree of persistence of the internal model after disembarkation. Normal synaptic plasticity could be converted into excessive synaptic plasticity by a predisposing factor expressed in some individuals. This is a point of convergence of our hypothesis with the theory of MdDS hormonal and neurochemical predisposition published in 2018 (7). As known, hormonal changes at the luteal phase of pre-menstrual syndrome, or in perimenopause, can influence the brain levels of CGRP, whose expression is regulated by gonadal hormones (7, 72, 73). CGRP has been reported as an LTP promoter (32), while its expression is closely correlated with that of the BDNF (31), which is the key element of bifurcation in the above-mentioned positive feedback loop that generates LTP (68).

**Figure 3:**
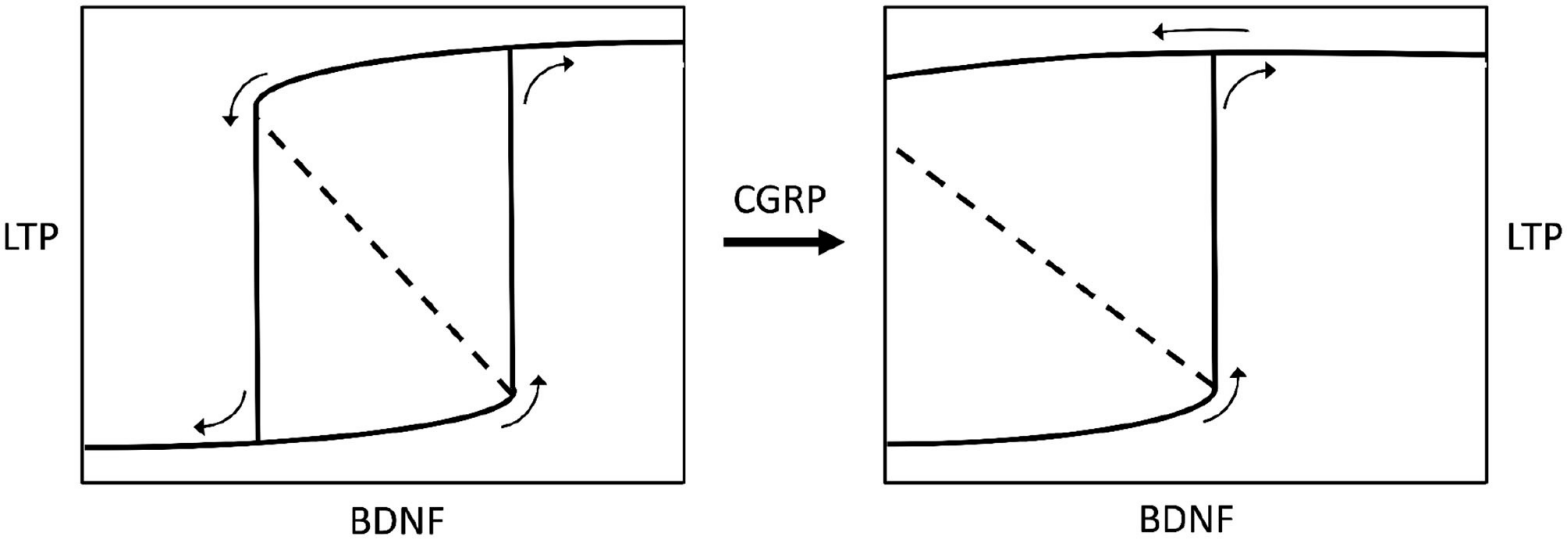
Bistable hysteretic model of synaptic plasticity depicted by a stimulus-response curve. The stimulus is represented by variations of brain-derived neurotrophic factor levels (BDNF) in the central nervous system. The response is a biochemical pathway arranged as a positive loop (not shown here) leading to synaptic long-term potentiation (LTP), according to Hao et al. (68). If the loop has a lower strength (left chart) the stimulus-response curve shows a bifurcation generating bistability and hysteresis. Bistability means the occurrence of two equilibrium points, i.e., low or high (excess) LTP. Hysteresis means that for some given values of BDNF there are two possible values of LTP, and the status assumed by the system will depend on its previous history, i.e., it will be low LTP for increasing BDNF, or high LTP for decreasing BDNF (see small arrows on charts). If the BDNF-induced, biochemical positive loop that produces LTP is progressively strengthened by calcitonin gene-related peptide (CGRP), the bistability region widens until hysteresis switches to irreversibility (right chart), meaning that once LTP is developed, after BDNF rise, it becomes permanent even if BDNF decreases to zero. The model has been envisaged according to data reported by Buldyrev et al. (31). Small arrows on charts indicate the evolution of the system for increasing or decreasing BDNF values.

BDNF is not only influenced by CGRP but also by gonadal hormones (74), possibly indicating that a particular hormonal state may induce neurochemical changes. In addition to this, CGRP is known to strengthen the VOR reflex (29), confirming the role of this neuromodulator in modifying the activity of vestibular neural networks, and being consistent with the correlation between CGRP brain levels and the severity of MdDS symptoms (34). Hence, by putting it all together, we can theorize that some gonadal imbalance, correlated with high CGRP brain levels, potentially affects other modulators, like BDNF, thereby leading to excessive synaptic plasticity and ultimately causing symptom chronicity in MdDS (Figure 3).

In summary, by our hypothesis MdDS is not only regarded as a maladaptation of the VOR, but upstream to it, as a malfunctioning of a vestibulocerebellar network that realizes an oscillatory loop system. According to this new approach, we have shown how this **loop system** could be entrained to oscillate by environment movements, thereby leading to MdDS through its over-synchronization. While undergoing synaptic plasticity, this process could become excessive in individuals experiencing an impairment in the brain levels of specific hormones and neuromodulators (*possibly including low levels of estrogen, as well as high levels of CGRP and BDNF*). This would render the internal oscillator noxiously persistent after disembarking from a vehicle, thereby leading to the constant perceptions of self-motion that we know as MdDS. This theory may also explain why MdDS patients experience a temporary relief of symptoms when exposed to passive motion, as mentioned before, because their over-synchronized loop would be working in its perfect status of canceling the external stimuli.

## Comparison With Previous Theories

In the herein proposed hypothesis, feedback loop dynamics are invoked as an explanatory umbrella for a series of ailments of increasing severity going from MdD to MdDS. The hypothesis is innovative in the framework of MdDS studies, but to some extent it is also a synthesis of different ideas that have been previously formulated about this syndrome. As discussed above, the most significant synergism can be established between our hypothesis and the hormone theory of MdDS (7). However, a close connection can also be found with the idea of an over-synchronization of brain networks caused by entrainment due to the exposure to passive motion (18, 21). The proposed treatments that follow this kind of analysis, such as transcranial Direct Current Stimulation (tDCS) (75) and repetitive Transcranial Magnetic Stimulation (rTMS) (21), are consistent with our hypothesis since they could disorganize an excessive neural network connection (21), but the success rate reported by patients is considered poor (22), suggesting that more refined targets and modalities of treatment are needed.

The theory concerning VOR and VS maladaptation (9, 76) is also somewhat consistent with our hypothesis, given that the neural circuits of these mechanisms are presumed to be, at least in part, coincident with our oscillating loop system. Following this theory, patients were exposed to a full-field optokinetic (OKN) stimulus during head rotation, obtaining an improvement of symptoms in 70% of cases (14), even though there was a slight decline in the success rate over time (9). Similar results were obtained in a recent study which involved a sham protocol for MdDS patients undergoing OKN stimulation, while it was speculated that OKN stimulation worked in re-adapting the so-called maladapted VOR (77). To date, the success rate of the OKN treatment is higher for those with MT MdDS compared to the SO patients (22), further research is needed to understand why this is the case. These studies considered the relationship between VOR and OKN reflexes, knowing that the VOR response can adapt and accommodate sensory arrangements, as shown in a study by Draper (78). The authors hypothesized that a disrupted VOR leads to a disrupted VS and VSR, which consequently leads to poor postural control (9). The results from this study (77) support Dai's theory that the OKN stimulation and head roll is able to induce a VOR adaptation process by altering the performance of the OKN reflex through visual anomalies. However, a striking difference with our hypothesis is that this VOR maladaptation would be a downstream consequence of MdDS, not a triggering effect, i.e., the cause-effect relationship between MdDS pathogenesis and VOR would be inverted. Nevertheless, the OKN treatment fits well also under our hypothesis, since a strong stimulation of the VOR could partially alleviate the excessive synaptic plasticity that we presume to be present in MdDS patients. In addition to this, as reported in the sham study (77), the OKN treatment could be just one part of the treatment process for MdDS subjects, since most subjects also continued to report associated symptoms such as migraine (7, 77) following a postural improvement.

## How to Validate the Hypothesis

Our hypothesis is, for now, only a theoretical model and a series of studies have to be developed and executed in order to validate it. Specific clinical protocols and tests will have to be developed according to a suitable, hypothesis-driven experimental design based on the herein proposed model, thus collecting data specifically suitable to prove or disprove the model.

One of the first aspects to consider is the assessment of the internal model, such as verifying that MdDS is triggered in the presence of a regular wave with a single (*or strongly dominant*) sinusoidal component (*boat or plane*); rather than in the presence of noise (*car or train*). Such a result has been partially achieved in a retrospective study on a large number of MdDS patients, showing that the disease is mainly triggered by boat travels (4). However, additionally, the mechanism could be further investigated with the use of computerized posturography tests under static or moving conditions at different frequencies of oscillations, to evaluate if subjective perception of motion matches with objective postural instability.

Secondly, to specifically prove the over-synchronization of an oscillatory neural loop system the use of neuroimaging techniques could be a suitable approach. By employing high-resolution functional magnetic resonance imaging (fMRI)/18F-fludeoxyglucose positron-emission tomography (18F-FDG–PET) scans, and electroencephalogram (EEG) it would be possible to explore variations in functional connectivity between VNs and the flocculonodular lobe. This kind of analysis could be done on controls and patients without previous treatment or after different exposures, including various kinds of vestibular stimulation, hence showing possible differences in vestibulocerebellar connectivity consistent with the hypothesized model. If the vestibulocerebellar connectivity pattern would show higher correlation in patients with respect to controls, selectively at the internal oscillator frequencies, this will confirm that the internal oscillator would have become undesirably persistent in MdDS. This would potentially provide a neuroimaging biomarker allowing to distinguish MdDS from other central vestibular disorders, especially SO MdDS, which might be falling more clearly into the PPPD diagnostic sphere.

In addition, as posed in the hypothesis, MdDS patients may develop this permanent form due to a neurochemical imbalance that would render them more susceptible to this maladaptation, hence their hormonal status and history should be taken into account (*e.g., perimenopausal, low testosterone or estrogen, and usage of steroids or hormonal replacement therapy)* (23).

Another method of exploring altered connections between VN and cerebellum, potentially affecting the functioning of VS, VOR, and other vestibular circuits, could be the use of transcranial magnetic stimulation (TMS), a technique that has contributed significantly to the understanding and treatment of several neurological and psychiatric disorders (79, 80). TMS can be used with single and paired pulses over the primary cortex (M1), allowing to trace excitatory and inhibitory pathways (79). For instance, paired-pulse TMS can induce short-interval intracortical inhibition (SICI) by involving GABA_A_ receptors (79, 81). TMS has been also used in combination with drugs (82), showing that benzodiazepine (*a positive modulator of GABA*_*A*_) enhances SICI (79), while long-interval intracortical inhibition (LICI) and cortical silent period (CSP), which are measures of long-lasting inhibition, increase with the GABA_B_ receptor agonists tiagabine (*working on* LICI) (83), and baclofen (*working on* CSP) (84). These techniques have been used to study the pathophysiology of motor system disorders such as amyotrophic lateral sclerosis, Parkinson's disease, Tourette syndrome, and altered motor cortex GABA_B_ function in concussed athletes (79, 80).

Despite that MdDS may not be a motor disorder, TMS could allow researchers to understand if a GABAergic alteration is characteristic of MdDS patients. Studying pharmacological changes with TMS allows for an indirect measure of excitatory and inhibitory mechanisms and their implications in neurotransmitters modulation. It has also been proposed that TMS could be used with *in vivo* proton magnetic resonance spectroscopy (1H-MRS), thus measuring the levels of GABA during different phases of the menstrual cycle and aligning these data with symptom intensity in MdDS patients (7). 1H-MRS would allow the detection and quantification of different neurometabolites besides GABA, such as myoinositol, N-acetylaspartate, and glutamate (79, 85).

A recent study utilized transcranial direct current stimulation (tDCS) to ease MdDS symptoms, with promising results (75). This novel neuromodulation technique would be advantageous for patients since it can be performed at home in a remote setting, reducing or eliminating long commutes which are known to cause discomfort for those with MdDS. Another technique used to test vestibular-spinal control system is Galvanic Vestibular Stimulation (GVS) (86). In a recent study on PPPD patients, GVS allowed the stimulation of vestibular afferents without head motion on either side separately, showing higher instability with higher intensity GVS (range from low to high, 0.8–2.8 mA) and closed eyes, consistent with the greater visual dependency in controlling posture of these subjects (86). A similar experiment could be performed on MdDS patients to examine their sensory reweighting.

Finally, the aforementioned postural, neuroendocrine, neurochemical, neuroimaging and transcranial stimulation data has the potential to contribute partial confirmations of our hypothesis, however their amassing has the ability to ultimately validate the hypothesis.

## Conclusions

MdDS remains a challenging problem for healthcare professionals. Due to unclear underlying mechanisms and the lack of definitive biomarkers, the diagnostic process is typically long and costly, while treatment options are experimental and limited. Our hypothesis provides an innovative vision into this syndrome, by proposing that dynamic loops are involved in brain adaptive responses to oscillatory passive motion. Our hypothesis does not reject previous theories on MdDS pathogenesis, but it rather embodies elements of these in a comprehensive mechanism, based on Systems and Control Theory. The main elements of our hypothesis are the following:

- Starting from an essential biomechanical analysis of posture, we derived the notion that perfect adaptation to an external oscillatory disturbance needs the **activation of an adaptation loop including an internal oscillator able to cancel disturbance** (Internal Model Principle).
- Thereafter, starting from available neuroanatomical and physiological knowledge of the vestibulocerebellar region we identified **a bilateral neural network arranged** as a **triple loop with two further contralateral connections**, involving the VNs and the cerebellar flocculonodular lobe.
- By using computational simulation, we proved the tendency of the **neural network to behave as an oscillator with the increasing strength of inhibitory connections**, reaching its highest when both inhibitory and excitatory connections are strong.
- We therefore assumed that the identified neural loop system becomes entrained by exposure to passive motion, e.g., those experienced onboard of a vehicle, thus activating an adaptive internal model.
- Finally, given that such entrainment is likely to involve synaptic LTP, and by assuming that synaptic plasticity is triggered by a biomolecular positive loop, we envisaged that under some unbalance of neuromodulators like CGRP and BDNF, during peculiar gonadal hormonal phases, the transition to LTP becomes irreversible. This would maintain the internal oscillator after the removal of the external stimulus (e.g., after disembarkation), thereby producing MdDS.

If this hypothesis were tested and verified, then optimal targets for MdDS treatment could be found inside the neural networks and biochemical factors that play a fundamental role in loop functioning and synaptic plasticity. We proposed a few studies to address this theory, such as: the use of dynamic posturography for exposing patients at different frequencies and evaluate their self-perception of motion; perform specific neuroimaging studies to explore variations in functional connectivity between VNs and the flocculonodular lobe in MdDS patients vs. healthy controls; assess patient's hormonal status and history and observe if specific hormonal imbalances or conditions are characterizing of MdDS patients; using TMS to study SICI and LICI by involving the use of medications and their effect on the patient's GABAergic system, evaluating if altered GABAergic system is present in MdDS patients, this could also be combined with 1H-MRS, thus measuring the levels of GABA during different phases of the menstrual cycle and aligning these data with symptom intensity in MdDS patients.

As a corollary, our hypothesis falls into a wider theory of the organism's physiology (67, 87), based on the assumption that dynamic loops are an essential trait for all processes and transitions that occur in the organism, notably transitions from physiological to pathological status. Therefore, this hypothesis can be taken as a basis for theoretical analysis and novel experimental models of other neurological disorders.

## Data Availability Statement

All datasets generated for this study are included in the article/Supplementary Materials.

## Author Contributions

VM is the primary author, she contributed to the formation of the team to collaborate and to the scientific basis for implementing the theory. II reviewed the hypothesis and contributed to the reviewing of the manuscript. CB reviewed the hypothesis and the manuscript. FB provided the mathematical model and contributed to the formation of the hypothesis. GG provided the mathematical model and contributed to the formation of the hypothesis. LM contributed to the validation of the hypothesis with clinical studies. BB contributed to the formation of the hypothesis and developed a pathophysiological model of MdDS. All authors contributed to the article and approved the submitted version.

## Conflict of Interest

The authors declare that the research was conducted in the absence of any commercial or financial relationships that could be construed as a potential conflict of interest.
