# Supplementary material for: Mal de Debarquement Syndrome: A Matter of Loops?
Source: Front Neurol. 2020 Nov 10;11:576860. doi: 10.3389/fneur.2020.576860 (PMC7683778; doi:10.3389/fneur.2020.576860)
Supplement: Supplementary file 1 [file Presentation_1.pdf]

The internal model mechanism
and its analysis

In this supplementary document, we provide a mathematical description of the internal model mechanism for posture stabilization and we analyze the resulting feedback system.

1 Biomechanical loops

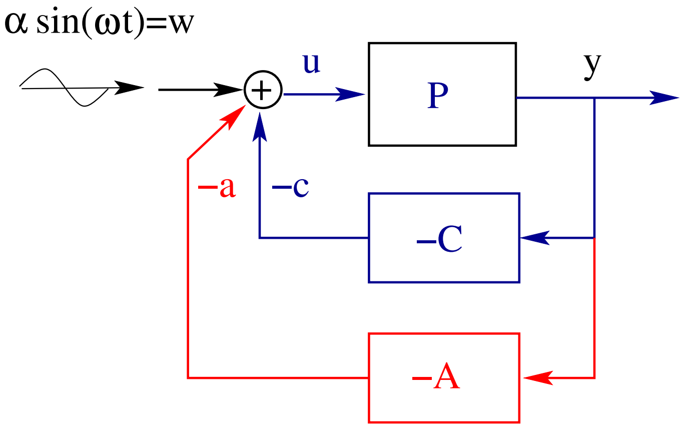

Figure 1: Posture control mechanism: biomechanical loops.

Consider the feedback scheme in Figure 1, where P represents the Posture, which is a naturally unstable mechanism, C the stabilizing posture control (negative feedback) and A an adaptation mechanism (negative feedback). We assume that the adaptation mechanism is activated when a sinusoidal disturbance of frequency $f = \omega/(2\pi)$ is applied to the system. The equations corresponding to the scheme are

$$\begin{aligned} u &= w - c - a \\ y &= Pu \\ c &= Cy \\ a &= Ay \end{aligned}$$

The "$-$" signs are conventionally included to recall that both feedback loops are negative. In the sequel, we denote by $\dot{f}(t)$ and $\ddot{f}(t)$ respectivey the first and the second time derivative of a function $f(t)$.

We adopt the linear representation of human posture, modeled as an inverted pendulum [1, 5]:

$$\ddot{y}(t) = \gamma y(t) + u(t).$$

Function y is the posture angle (the angle between body axis and gravity) oriented in the upward direction ($y = 0$ is the target). This simplified representation is widely accepted as a faithful representation of human posture, at least for small perturbations, as discussed in [4]. Posture is, of course, an unstable mechanism that requires stabilization. Consistently with existing work, we assume that the stabilizing mechanism is of the Proportional-Derivative type [1, 4, 5]:

$$c(t) = \rho(y(t) + \theta\dot{y}(t)).$$

The parameter ρ is the feedback intensity, while θ is the damping coefficient, which establishes the intensity of the action proportional to the speed. There are delays in this loop [1]; since these amount to a few milliseconds, they are neglected in our investigation, but our study can be extended without essential changes to the case of delays.

To analyze the system, we adopt a linear analysis, based on the Laplace transform. This means that we symbolically replace the derivative by the s operator, so that $\dot{y} \to sy$ and $\ddot{y} \to s^2 y$. We get $s^2 y = \gamma y + u$, or equivalently

$$y = \frac{1}{s^2 - \gamma} u = P(s)u. \tag{1}$$

Function $P(s) = \frac{1}{s^2-\gamma}$ is called the transfer function. All the components in the scheme can be represented by suitable transfer functions: $P(s)$ represents posture, $C(s)$ the compensator, and $A(s)$ the adaptation mechanism. In particular, the transfer function of the posture feedback stabilization is $C(s) = \rho(1 + \theta s)$, precisely

$$c = \rho(1 + \theta s)y = C(s)y. \tag{2}$$

After simple computations, by replacing into (1) the expression of $u = w - a - c$ and replacing c with its expression given in (2), we get

$$y = \frac{1}{s^2 + \rho\theta s + (\rho - \gamma)}(w - a) = F(s)(w - a).$$

The stability condition for this system is $\rho > \gamma$; let us assume that the stability condition is satisfied. In the absence of disturbance, i.e. $w = 0$, no adaptation is necessary (hence $a = 0$) and the posture variable y converges to its target value 0. However, in the presence of a persistent perturbation

$$w = \alpha \sin(\omega t),$$

the rest condition cannot be ensured without the adaptation signal a, which attenuates the effects of, and possibly even erases, the disturbing signal w.

Perfect adaptation requires the ideal condition that a cancels w:

$$a + w = 0.$$

The signal a opposing w must be: (p1) synchronized with w; and (p2) of the same amplitude α of w, which is known. To ensure properties (p1) and (p2), the adaptation signal must be produced by a feedback mechanism $a = A(s)y$. After simple computations, the overall mechanism becomes

$$y = \frac{A^{-1}(s)F(s)}{A^{-1}(s) + F(s)}w = W(s)w$$

The following properties are relevant to our study. These hold in general for systems represented by a generic transfer function $T(s)$.

Proposition 1 *Given the periodic signal $w = \alpha \sin(\omega t)$ of frequency $f = \omega/(2\pi)$, the output y of the stable system represented by the transfer function $T(s)$ converges to zero, despite the disturbance w, if and only if we have $T(s) = T(j\omega) = 0$ for $s = j\omega$.*

Proposition 2 *The system represented by the transfer function $T(s)$ is an oscillator that produces persistent oscillations (under stability assumptions), of frequency $f = \omega/(2\pi)$, if $T(j\omega)^{-1} = 0$.*

We can apply these properties to our case, considering the resulting transfer function W as follows.

- If $W(j\omega) = 0$, then the system $y = W(s)w$ cancels any sinusoidal signal w at frequency $f = \omega/(2\pi)$. This is may be achieved only when $A(j\omega)^{-1} = 0$, because $F(j\omega) \neq 0$.
- In turn, since we must have $A(j\omega)^{-1} = 0$, then the system $A(s)$ is an oscillator, which produces a sinusoidal signal a at frequency $f = \omega/(2\pi)$ even without external inputs.

To proceed in the investigation, we consider the adaptation feedback $A(s)$ to be of the form

$$A(s) = \frac{\kappa(1+\tau s)}{s^2+\omega^2}, \qquad \text{hence} \qquad A(s)^{-1} = \frac{s^2+\omega^2}{\kappa(1+\tau s)}. \tag{3}$$

Therefore, $A(s)$ is the joint action of the term $\frac{1}{s^2+\omega^2}$, which is an oscillator, and the term $\kappa(1+\tau s)$, which is again a Proportional-Derivative controller, where κ is the feedback intensity and τ is the damping factor. This simple adaptation mechanism can properly cancel the external disturbance. Actually, among all possible mechanisms that can properly perform the job, this one is the simplest in terms of the degree of A (which is of the second order, the minimum order required to be an oscillator).

If we plug the obtained expression of A into the expression of W, we get

$$W(s) = \frac{s^2+\omega^2}{(s^2+\theta\rho s+(\rho-\gamma))(s^2+\omega^2)+\kappa(1+\tau s)}$$

For this closed-loop transfer function, the condition $W(j\omega) = 0$ is satisfied, hence perfect adaptation is achieved. It is important to stress that there is no other way to have perfect adaptation: the dynamical system associated with $A(s)$ needs to be an undamped oscillator, because the signal a must cancel the signal w, hence it must be periodic so as to ensure $a + w = 0$.

This property is the so-called *internal model principle*. The terminology is due to the following interpretation: the wave signal w is assumed to be produced by an "external sinusoidal generator" (for instance, the sea in the case of waves moving a boat and inducing motion sickness) and, to be successful in canceling the external disturbance, the adaptation mechanism must include a proper "model" that reproduces such an external generator.

Proposition 3 *Assume that a system $F(s)$ does not have the natural property of perfectly rejecting a signal at frequency $\omega/(2\pi) = f$, namely $F(j\omega) \neq 0$. Then, any feedback loop capable of ensuring perfect adaptation to a signal $w = \rho \sin(\omega t)$ must include an internal model, namely a device able to generate a signal at the same frequency f.*

2 Analysis of the loop without the forcing term

The next step is the analysis of what happens when the persistent signal is removed (e.g. in the case of disembarkment), i.e. $w = 0$. Under normal conditions, the adaptation mechanism is interrupted almost immediately when the forcing term is absent. Conversely, if the internal oscillator that had been previously activated remains active, under particular conditions, it can therefore cause a feeling of dizziness [2, 3].

We wish to explain the phenomenon from a control theory standpoint via mathematical loop analysis. Even in the presence of adaptation, the mechanism is stable. So why does the oscillatory pattern remain? This can be explained by the presence of poorly damped oscillating modes.

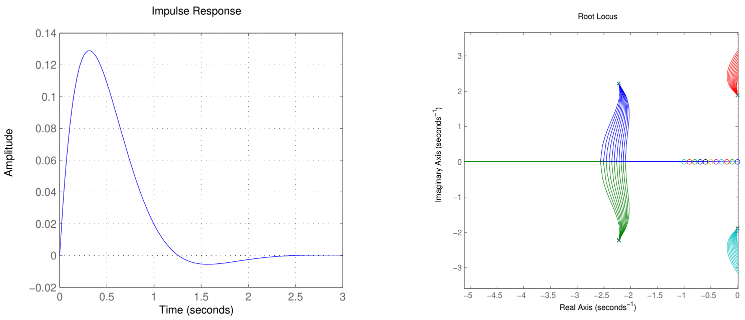

Figure 2: The posture controlled system. Left: reaction to an impulse perturbation (e.g. an abrupt hit), with no adaptation mechanism; the reaction time is of about 1 s. Right: the root locus for all possible stabilizing values of the parameters κ and τ. There are two complex roots close to the imaginary axis, which reveal the presence of poorly damped oscillations that are slow at disappearing.

The parameters of the stabilizing feedback ρ, θ and c are typically unknown and difficult to estimate. Therefore, we directly consider the transfer function F of the system that incorporates the stabilizing feedback and we rewrite it in the parametric form

$$F = \frac{1}{s^2 + \rho\theta s + (\rho - c)} = \frac{1}{s^2 + 2\xi\lambda s + \lambda^2}$$

where ξ and λ should to be estimated from postural experiments monitoring the reaction time of a person standing and subject to an abrupt hit.

While the damping factor ξ can be reasonably set to $\xi = 1/\sqrt{2}$, λ is associated with the reaction time to a posture perturbation (impulse response, essentially the response to a hit). It is also associated with the critical frequency associated to the stabilized posture [5]. Considering a frequency 0.5, we derive the value $\lambda = 3.14$, corresponding to a setting time of about 2 seconds, as

in Figure 2 (left). In general ξ and λ depend on the weight and height of the subject (the larger these parameters, the lower the frequency) and on health conditions.

The closed-loop system with the adaptation mechanism is *stable*, but nevertheless it exhibits poorly damped oscillations. These oscillations are explained by the presence of two complex roots of the closed-loop system that are close to the imaginary axis and have a frequency very close to $f = \omega/(2\pi)$, as shown next.

How can we estimate the parameters for A? The parameter ω is fixed by the frequency f of the disturbance: $\omega = 2\pi f$. Here, we have chosen a frequency $f = 0.3$, corresponding to $\omega = 1.8850$. Unfortunately, neither κ not τ are available and measuring them is hopeless.

We therefore resort to a parametric investigation: we consider all possible values of $\kappa > 0$ and $\tau > 0$. The results can be seen from the envelope of the root locus in Fig. 2 (left). For all choices of the parameters $\kappa > 0$ and $\tau > 0$ the closed loop system has a complex pair of poles that are close to the imaginary axis and have an imaginary part close to ω. These are included in the two specular red and cyan regions on the right in the panel.

These roots are associated with the poorly damped oscillations that explain the feeling of dizziness. The impulse response in the presence of the adaptation mechanism is shown in Fig. 3, and as expected exhibits poorly damped oscillations that slowly fade.

Note that, although slowly, these poorly damped oscillations are very likely to fade out in a reasonably short time; to explain why oscillations can become *persistent* and lead to MdDS, the role of excessive synaptic plasticity is discussed in the main paper.

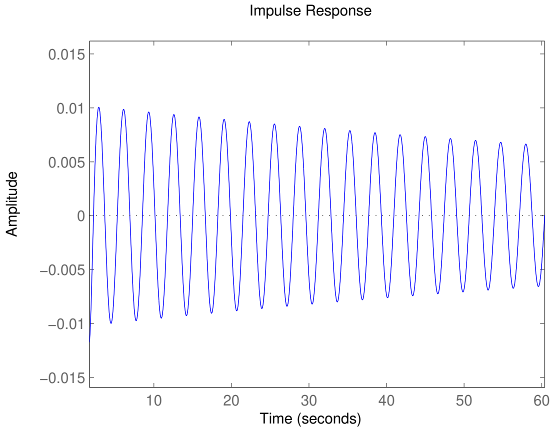

Figure 3: The impulse response in the presence of the adaptation mechanism, showing poorly damped oscillations.

3 Conclusions

The previous considerations lead to the following conclusions.

- Human posture corresponds to an unstable system modeled as an inverted pendulum, which requires a stabilizing feedback.

- To achieve adaptation to an external sinusoidal disturbance, an additional feedback action is necessarily involved: we call it *adaptation mechanism*.
- The adaptation is perfect if the loop includes an oscillator capable of generating signals having the same frequency as the disturbance signal.
- The mechanism is specific for a certain frequency.
- The inclusion of the adaptation mechanism in the loop introduces a poorly damped oscillation, at a frequency equal or close to that of the disturbance.
- The mechanism might (temporarily) persist even after the disturbance has been removed, thus explaining dizziness.

These considerations support the idea that the MdD is explained by the undesired persistence of the adaptation mechanism as argued in [2, 3].

References

[1] Alexandrov AV, Frolov AA, Horak FB, Carlson-Kuhta P, Park S, "Feedback equilibrium control during human standing", *Biol. Cybern.* (2005) 93: 309–322.

[2] Hain TC, Helminski JO. Mal de Debarquement. In "*Vestibular Rehabilitation*", 2nd edn (Ed. S. Herdman), 2007.

[3] Hain TC, *Mal de Debarquement Syndrome (MdDS or MdDS)* November 28, 2019. https://www.dizziness-and-balance.com/disorders/central/mdd.html

[4] Morasso P, Cherif A, Zenzeri J, "Quiet standing: The Single Inverted Pendulum model is not so bad after all", *PLOS ONE* (2019) 14(3): e0213870. https://doi.org/10.1371/journal.pone.0213870.

[5] Terekhov AV, Levik YS, Solopova IA, "Mechanisms of reference posture correction in the system of upright posture control", *Human Physiology* (2007), 33:289–295.

Mathematical analysis of the vestibulocerebellar loop system

In this supplementary document, we provide a mathematical description of the dynamic evolution of the key players involved in the feedback loop system shown in Fig. 2A.

To this aim, we denote the activities of the functional agents (neurons) as follows:

P_L = Purkinje neurons to the left of brainstem midline;
P_R = Purkinje neurons to the right of brainstem midline;
VN_L = vestibular nuclei to the left of brainstem midline;
VN_R = vestibular nuclei to the right of brainstem midline.

In addition, we assume that each of the above functional agents, say x, is subject to a spontaneous reduction of its activity and evolves with time constant τ_x.

The activating (blue) and inhibitory (red) interactions visualized in Fig. 2A are modelled in terms of monotonic functions. In particular, we denote by f an activation function, which is monotonically increasing in its argument, and by g an inhibition function, which is monotonically decreasing in its argument (possible examples, just for illustrative purposes, are the well-known Hill functions having expression $f(x) = \frac{\alpha x^p}{1+\beta x^p}$ and $g(x) = \frac{\gamma}{1+\delta x^p}$, where the integer p is the Hill coefficient and the Greek letters are positive real parameters).

Then, the dynamics associated with the feedback loop arrangement visualized in Fig. 2A is described by the following system of ordinary differential equations:

$$\tau_{P_L}\dot{P}_L + P_L = f_1(VN_L) + g_1(VN_R) \quad (1)$$
$$\tau_{VN_L}\dot{VN}_L + VN_L = g_2(P_L) + g_3(VN_R) \quad (2)$$
$$\tau_{VN_R}\dot{VN}_R + VN_R = g_4(VN_L) + g_5(P_R) \quad (3)$$
$$\tau_{P_R}\dot{P}_R + P_R = f_2(VN_R) + g_6(VN_L) \quad (4)$$

where $\dot{x}$ denotes the time derivative of x.

Let us define the positive quantities:

$\alpha = \frac{1}{\tau_{P_L}}$,
$\beta = \frac{1}{\tau_{VN_L}}$,
$\gamma = \frac{1}{\tau_{VN_R}}$,
$\delta = \frac{1}{\tau_{P_R}}$,
$\mu = \frac{1}{\tau_{P_L}} \frac{\partial f_1(VN_L)}{\partial VN_L}$,

$\nu = -\frac{1}{\tau_{P_L}} \frac{\partial g_1(VN_R)}{\partial VN_R}$,
$\kappa_1 = -\frac{1}{\tau_{VN_L}} \frac{\partial g_2(P_L)}{\partial P_L}$,
$\sigma = -\frac{1}{\tau_{VN_L}} \frac{\partial g_3(VN_R)}{\partial VN_R}$,
$\varphi = -\frac{1}{\tau_{VN_R}} \frac{\partial g_4(VN_L)}{\partial VN_L}$,
$\kappa_2 = -\frac{1}{\tau_{VN_R}} \frac{\partial g_5(P_R)}{\partial P_R}$,
$\tau = -\frac{1}{\tau_{P_R}} \frac{\partial g_6(VN_L)}{\partial VN_L}$
and
$\xi = \frac{1}{\tau_{P_R}} \frac{\partial f_2(VN_R)}{\partial VN_R}$.

Then, the Jacobian matrix of the system can be written as:

$$J = \begin{bmatrix} -\alpha & \mu & -\nu & 0 \\ -\kappa_1 & -\beta & -\sigma & 0 \\ 0 & -\varphi & -\gamma & -\kappa_2 \\ 0 & -\tau & \xi & -\delta \end{bmatrix}$$

where the diagonal entries $(-\alpha, -\beta, -\gamma, -\delta)$ are associated with self-inhibitory connections, the positive off-diagonal entries (μ, ξ) are associated with excitatory connections, and the negative off-diagonal entries $(-\nu, -\kappa_1, -\sigma, -\varphi, -\kappa_2, -\tau)$ are associated with inhibitory connections.

We wish to understand whether the associated dynamical system can yield persistent oscillations; to this aim, the Jacobian matrix J needs to have strictly complex eigenvalues with positive real part.
